# The Others: A Systematic Review of the Lesser-Known Arboviruses of the Insular Caribbean

**DOI:** 10.3390/v15040843

**Published:** 2023-03-25

**Authors:** Inshan Ali, Pedro M. Alarcόn-Elbal, Marcia Mundle, Simmoy A. A. Noble, Chris A. L. Oura, Joshua J. Anzinger, Simone L. Sandiford

**Affiliations:** 1 Department of Microbiology, Faculty of Medical Sciences, The University of the West Indies, Mona, Kingston 7, Jamaica; 2 Department of Animal Production and Health, Veterinary Public Health and Food Science and Technology (PASAPTA), Facultad de Veterinaria, Universidad Cardenal Herrera-CEU, CEU Universities, 46115 Valencia, Spain; 3 Department of Natural Sciences, Faculty of Science and Technology, The Mico University College, Kingston 5, Jamaica; 4 School of Veterinary Medicine, Faculty of Medical Sciences, The University of the West Indies, St. Augustine 685509, Trinidad and Tobago; 5 Global Virus Network, Baltimore, MD 21201, USA; 6 Department of Basic Medical Sciences, Faculty of Medical Sciences, The University of the West Indies, Mona, Kingston 7, Jamaica; 7 Mosquito Control and Research Unit, The University of the West Indies, Mona, Kingston 7, Jamaica

**Keywords:** Caribbean, arboviruses, One Health, hotspot, hematophagous arthropods

## Abstract

The Caribbean enjoys a long-standing eminence as a popular tourist destination; however, over the years it has also amassed the sobriquet “arbovirus hotspot”. As the planet warms and vectors expand their habitats, a cognizant working knowledge of the lesser-known arboviruses and the factors that influence their emergence and resurgence becomes essential. The extant literature on Caribbean arboviruses is spread across decades of published literature and is quite often difficult to access, and, in some cases, is obsolete. Here, we look at the lesser-known arboviruses of the insular Caribbean and examine some of the drivers for their emergence and resurgence. We searched the scientific literature databases PubMed and Google Scholar for peer-reviewed literature as well as scholarly reports. We included articles and reports that describe works resulting in serological evidence of the presence of arboviruses and/or arbovirus isolations in the insular Caribbean. Studies without serological evidence and/or arbovirus isolations as well as those including dengue, chikungunya, Zika, and yellow fever were excluded. Of the 545 articles identified, 122 met the inclusion criteria. A total of 42 arboviruses were identified in the literature. These arboviruses and the drivers that affect their emergence/resurgence are discussed.

## 1. Introduction

It is estimated that more than 80% of the world’s population inhabits areas at risk of vector-borne diseases, the most predominant being arboviruses, an acronym derived from “arthropod-borne virus” [1]. Arboviruses are maintained in nature through biological transmission between susceptible vertebrate hosts and hematophagous arthropods [2] such as mosquitoes (Diptera: Culicidae), biting midges (Diptera: Ceratopogonidae), sandflies (Diptera: Psychodidae), and ticks (Ixodida: Ixodidae, Argasidae), with mosquitoes being, by far, the most relevant. Despite not comprising a formal virologic taxonomic group, the arboviruses share several clinical and epidemiologic similarities as well as virologic characteristics, such as an RNA genome [3], with the exception of African swine fever virus. There are currently 537 arboviruses registered in the International Catalogue of Arboviruses, and this estimate is continuously increasing as advances in virus isolation procedures and sequencing methods impact virus studies. Many of these viruses do not appear to be human or animal pathogens; however, more than 130 are recognized as causing mild-to-fulminant disease in humans [4].

**Figure 1:**
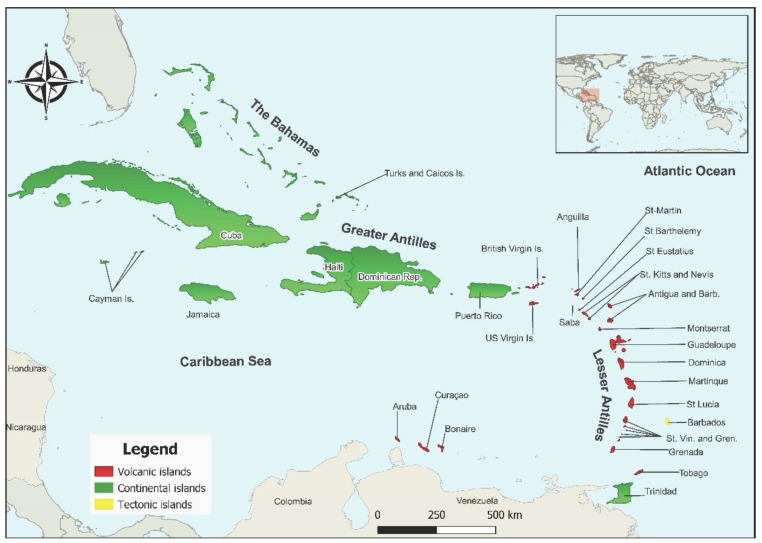
Map highlighting the geological history of the islands of the Caribbean. The Caribbean is composed of three island groups—The Bahamas, Greater Antilles, and Lesser Antilles. Abbreviations: St. Vincent and the Grenadines (St. Vin. and Gren.); Dominican Republic (Dominican Rep.) Antigua and Barbuda (Antigua and Barb.). Map created using QGIS 3.28.1.

There are over 7000 islands of the insular Caribbean, which covers an area of 224,570 km^2^ from the southern tip of Florida to northeastern Venezuela. It is composed of three island groups: The Bahamas; the Greater Antilles, consisting of the larger islands of Cuba, Hispaniola (divided between Haiti in the west and the Dominican Republic in the east), Jamaica, and Puerto Rico; and the Lesser Antilles, comprising the smaller islands [5]. Though commonly united by the ocean, they do not all share the same geological history, as illustrated in Figure 1. Some are continental islands, which are land masses that were once connected to a continent, such as those belonging to the Greater Antilles and Trinidad. Others are tectonic islands, resulting from upward movement of material as earth plates scrape against each other. Volcanic islands result from the eruption of oceanic volcanoes with the resulting buildup of lava and ash, while coral islands are low, flat islands comprising mainly coral reefs [6,7]. These variances in geological evolution assist in explaining disparities in the biodiversity of their respective flora and fauna. As such, there are large differences in the abundance of vector species and reservoir hosts amongst the islands of the insular Caribbean, which may influence the numbers of arboviruses identified on any given island. However, many other drivers contribute to the emergence of arboviral diseases, such as human movement, and anthropogenic and climate changes [8].

It is well known that trade, travel, and migration are all contributors to the emergence and spread of vectors and arboviral diseases [9]. We have seen how the used tire trade facilitated the introduction of *Aedes albopictus* to the Western Hemisphere and its subsequent spread in the Caribbean [10,11]. Similarly, tourism, being the economic backbone of most Caribbean islands, remains one of the main drivers of human movement, and there have been numerous reports of travelers returning to their home countries with arbovirus infections [12,13,14,15]. Natural disasters can also result in mass movements of people, and it is estimated that a person living in a small island developing state (SIDS) is three times more likely to be displaced by a natural disaster compared to a person living elsewhere [16]. Additionally, anthropogenic changes may also influence the way in which humans interact with reservoir hosts and vectors, thus modifying potentials for disease emergence. For instance, urbanization is associated with strongly reduced species diversity and the possible domestication of vector species, as was seen with *Aedes aegypti* and dengue fever [17,18]. Agricultural expansion may also displace some vectors whilst creating favorable conditions for others, which, along with livestock intensification, can inflate vector population sizes and disease epidemiology [19]. Furthermore, climate change has become increasingly important in the emergence of arboviral diseases, and the Caribbean has reported rising temperatures between the years 1961 and 2010, regionwide [20]. Such temperature changes have been shown to increase egg viability and vector competence [21,22] whilst decreasing egg hatching times and rates of viral replication within mosquitoes [23,24].

As the image of the insular Caribbean as an interconnected region rather than isolated islands becomes clearer, the propensity for dissemination of arboviruses and their vectors both regionally and globally becomes more apparent. Arboviruses such as dengue, chikungunya, Zika and yellow fever viruses have been adequately discussed elsewhere and are referenced for further reading [25,26,27,28]. This review aims to provide a current overview of lesser-known arboviruses identified in the Caribbean, highlight the drivers for emergence and resurgence of arboviral diseases, and emphasize the importance of the One Health approach in addressing the threats both regionally and globally.

## 2. Materials and Methods

For the purpose of this review, we examined evidence for the presence of arboviruses in the insular Caribbean. These include the islands of The Bahamas, those of the Greater Antilles (Cayman Islands, Cuba, Hispaniola, Jamaica, Puerto Rico), and those of the Lesser Antilles (Anguilla, Antigua and Barbuda, Aruba, Barbados, British Virgin Islands, Curaçao, Grenada, Guadeloupe, Martinique, Montserrat, Saba, St. Barts, St. Eustatius, St. Lucia, St. Kitts and Nevis, St. Martin, St. Vincent and the Grenadines, Trinidad and Tobago, United States Virgin Islands). The Preferred Reporting Items for Systematic Reviews and Meta-Analyses (PRISMA) guidelines were adopted [29]. 

### 2.1. Search Strategy

To ensure a comprehensive evaluation of the literature, we searched the scientific databases PubMed (https://pubmed.ncbi.nlm.nih.gov/ (accessed on 28 December 2022)) and Google Scholar (https://scholar.google.com/ (accessed on 28 December 2022)) for peer-reviewed literature as well as scholarly reports. Key words used to initiate searches in PubMed included “arbovirus”, “arbovirus Caribbean” “arbovirus in (specific island)”, “(specific) arbovirus in the Caribbean”, and in some cases, “(specific) arbovirus in (specific island)”, and in Google Scholar, “arbovirus insular Caribbean”.

### 2.2. Selection Criteria

Inclusion criteria were primary and secondary articles as well as scholarly reports that included works resulting in serological evidence and/or arbovirus isolations in the insular Caribbean. Articles and reports that involved dengue, chikungunya, Zika, and yellow fever were excluded. Arbovirus studies without serological evidence and/or arbovirus isolations in the insular Caribbean were also excluded. This review primarily focused on English language papers, but also included literature published in Spanish and Russian. All attempts were made to include the earliest available reports and publications and retrieve the literature available in the public domain.

### 2.3. Screening and Data Extraction

Study selection was carried out in three phases: identification, screening, and eligibility. Two independent researchers conducted the screening of the articles, with a third researcher solving any disagreements.

## 3. Results

### 3.1. Study Selection

**Figure 2:**
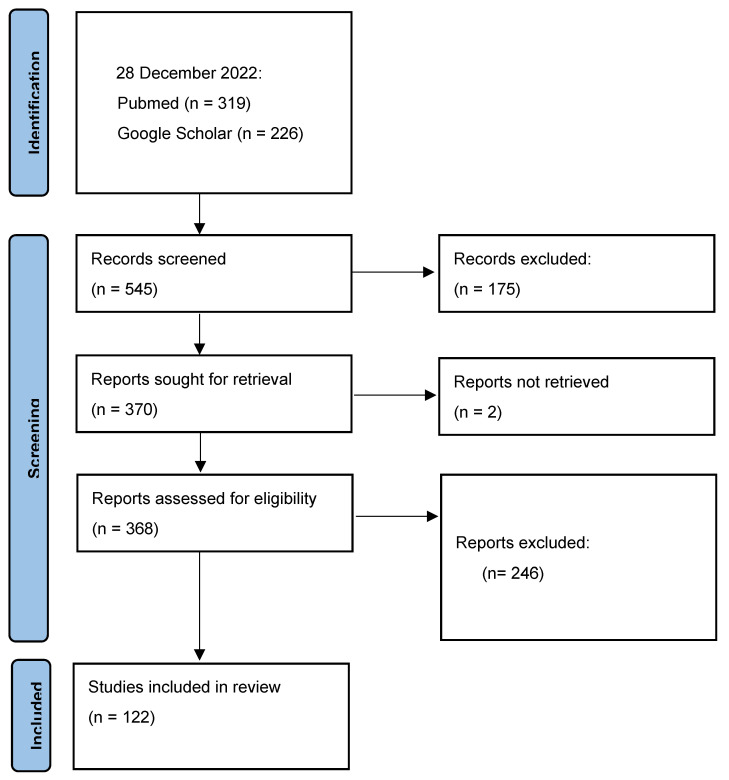
Preferred Reporting Items for Systematic Reviews and Meta-Analyses (PRISMA) flow chart.

Our electronic search identified 545 articles for potential screening. After removing duplicates and papers that did not contain evidence for the presence of arboviruses in the insular Caribbean, 370 studies remained for title/abstract examination and further screening. Two articles were not able to be sourced, and a total of 246 were excluded as they did not meet the inclusion criteria, with 122 finally included for the full review (Figure 2).

### 3.2. Sample Characteristics

Of the 122 articles included for the full review, 112 were primary sources, 7 were secondary sources, and 3 were scholarly reports. The majority of studies involved virus isolations exclusively from vectors. Most were from mosquitoes (*n* = 16) [30,31,32,33,34,35,36,37,38,39,40,41,42,43,44,45], with a minority involving ticks (*n* = 3) [46,47,48] and one from biting midges [49]. Few reports of isolations from humans were recorded (*n* = 7) [50,51,52,53,54,55,56,57,58]. The remaining studies involved isolations from combinations of vectors and animals as well as serological evidence from animals and humans.

### 3.3. Lesser-Known Arboviruses Identified from the Insular Caribbean

Arboviruses affecting human and animal health that have been identified in the Caribbean are discussed, and these span the following families: Asfaviridae, Flaviviridae, Nairoviridae, Peribunyaviridae, Phenuiviridae, Rhabdoviridae, Sedoreoviridae, and Togaviridae. Below is a brief introduction to each virus family and a summary overview of the known arboviruses in the region.

#### 3.3.1. Asfaviridae

The single species of this family is African swine fever virus (ASFV), which possesses a linear dsDNA genome and primarily affects swine. Infection is transmitted by various means, including via ticks of the genus *Ornithodoros* [59].

##### African Swine Fever Virus (ASFV)

The first reported outbreak of African swine fever (ASF) in the Western Hemisphere occurred in Cuba in 1971, resulting in the slaughter of 500,000 pigs [60]. This was followed by confirmation of the disease in the Dominican Republic in 1978 [61]. After slaughtering of pigs and the introduction of sentinel animals, up to 1981 negative cases of clinical disease were recorded, and all serological tests were negative [62]. The ASFV was ultimately identified in neighboring Haiti in 1979, leading to nationwide spread [61] and eventual eradication in 1982 [63]. Recently, in 2021, ASFV was detected in samples collected from pigs in the Dominican Republic [64] and a positive case of ASF was also confirmed in Haiti [65]. ASFV is continuing to spread in both Haiti and the Dominican Republic at the time of writing this review article.

#### 3.3.2. Flaviviridae

This family consists of enveloped, positive-sense non-segmented RNA viruses that are primarily spread through arthropod vectors infecting birds and mammals [66].

##### Ilhéus Virus (ILHV)

Inconclusive evidence of ILHV was reported from serological surveys conducted on multiple islands in the 1950s using hemagglutination inhibition (HI), the neutralization test (NT), and the complement fixation (CF) test. In most cases, sera also reacted with dengue virus (DENV), which confounded data interpretation. As such, no definitive conclusions regarding the presence of ILHV could be drawn from studies conducted in Antigua [67], Curaçao [68,69], Grenada [70], St. Lucia [71], or St. Vincent [72]. Only on the island of Trinidad was serological evidence of ILHV supported by viral isolations from three different mosquito species and humans by intracranial inoculation of mice [50,73,74] (Table 1).

##### Japanese Encephalitis Virus (JEV)

A single study conducted in Curaçao in 1953 reported the detection of JEV antibodies in the sera of convalescent patients with encephalitis. As sera were also positive for other antigenically related neurotrophic arboviruses, the authors noted difficulties with serological interpretations from this study [75].

##### St Louis Encephalitis Virus (SLEV)

Although widely reported throughout the region, evidence supporting the presence of SLEV in the insular Caribbean varies considerably. In some instances, only human serological data from single studies using HI, NT, and CF assays were reported. The authors caution interpretation of the results from work conducted in Antigua [67], Aruba [68], Barbados [76], Curaçao [68,69,75], Grenada [70], Montserrat [77], and St. Vincent [72]. In other islands, avian studies dominate the literature, as is the case for the Dominican Republic [78] and Puerto Rico where evidence of SLEV infections in birds was illustrated by the plaque reduction neutralization test (PRNT) [79,81]. In Cuba, multiple studies were conducted, and serological evidence provided from humans, horses, and birds using HI and NT indicated the presence of SLEV [82,83,84].

The most compelling data come from countries reporting viral isolations in addition to serological evidence (Table 1). In Haiti, SLEV was isolated in 1955 from a bird [85], and a subsequent vertebrate study conducted in 1972 identified neutralizing antibodies against SLEV in resident bird species [86]. Isolations in Jamaica occurred from mosquitoes and a bird in 1962–1963 [87,88,89]. Serological evidence preceded viral isolation in Jamaica, as unpublished reports of SLEV have existed since 1952 [90]; however, its widespread presence in humans was not confirmed until a 1956 serological study using HI and NT [91]. Clinical reports were also noted, as three human cases of acute viral encephalitis from the period of 1952–1961 were attributed to SLEV [92]. Additional serological evidence between 1960 and 1963 from humans, birds, and horses using HI and NT again confirmed its presence throughout the country [48], and more recently, avian studies using PRNT detected SLEV in resident bird species [79]. Similarly, the first evidence in Trinidad was provided during an island-wide serological survey during the period 1953 to 1954 [73]. Subsequently, from 1955 to 1962 multiple viral isolates were retrieved from mosquitoes, birds, and a human [93,94,95,96]. Recent serological studies conducted between 2006 and 2009 found evidence of SLEV in bats, horses, livestock, and wildlife [97,98].

##### Spondweni Virus (SPONV)

Thus far, SPONV has only been detected in Haiti. The virus was identified from a mixed-sex pool of mosquitoes collected in 2016. Initial rRT-PCR results suggested ZIKV, but unsuccessful attempts at amplification using ZIKV-specific primers lead to an unbiased sequencing approach and the identification of SPONV [30] (Table 1).

##### West Nile Virus (WNV)

Although the presence of WNV has been reported from many islands, few studies have demonstrated the presence of human infections in the insular Caribbean. The first confirmed human case occurred in 2001 and involved a resident of Cayman Brac who was admitted to hospital with viral encephalitis; WNV was confirmed by PRNT [99,100]. In 2003, West Nile neuroinvasive disease was observed in a patient in the Bahamas [101], and WNV was confirmed serologically in humans with encephalitis in Cuba between 2003 and 2004 [83]. In 2004, two patients with acute WNV infections were identified during mosquito-borne disease surveillance in Haiti using a microsphere-based immunoassay (MIA) and PRNT [102]. Although no additional human cases were reported, further studies conducted in Haiti in 2013 using an IgG ELISA showed that the seroprevalence of anti-WNV IgG antibodies in the tested population was 1% [103]. In Puerto Rico, evidence for WNV circulation was described in 2004 via ELISA and PRNT confirmation in nonmigratory native Puerto Rican birds [79]. Isolation of the virus using serological and molecular techniques occurred in 2006 from sentinel chickens and mosquitoes [104] (Table 1). In 2007, WNV was also identified by RT-PCR in human blood donors, birds, and mosquitoes [101,105]. Other studies similarly identified avian hosts by ELISA and PRNT testing [81], and there was serological evidence of WNV infection in horses [101]. Molecular evidence also existed in the British Virgin Islands, where PCR tests detected WNV from a formalin-fixed, paraffin-embedded heart of a flamingo chick [106]. In all other countries, only serological data from various species have been provided. Antibodies to WNV were detected in multiple hosts in Cuba [79,107], birds in the Dominican Republic [78,108], multiple hosts in Guadeloupe [109,110], birds in Jamaica [80], donkeys in St. Eustatius [111], equines in St. Kitts and Nevis [111], and multiple hosts in Trinidad [98,112].

#### 3.3.3. Nairoviridae

Members of this family are enveloped, negative-sense RNA viruses with three single-stranded RNA segments: S, M, and L. Viruses are transmitted by ticks to mammals and birds [113].

##### Estero Real Virus (ERV)

In 1980, eight antigenically similar strains of a new viral agent were isolated from ticks that parasitized bats collected in Cuba [46] (Table 1).

##### Hughes Virus (HUGV)

Multiple isolations of HUGV were made from two different islands in the Caribbean (Table 1). Between 1962 and 1965, HUGV was recovered in Trinidad from ticks collected feeding on tern chicks, and from nestling Sooty Terns [114], and in Cuba, from 1979 and 1980, from ticks [115,116].

##### Soldado Virus (SOLV)

Ticks and bird sera were collected from Soldado Rock in Trinidad between 1961 and 1965. Soldado virus was isolated from a pool of tick nymphs that was collected in 1963 and inoculated into two-day-old Swiss mice, which resulted in the death of one mouse [47] (Table 1).

#### 3.3.4. Peribunyaviridae

Viruses in this family are enveloped and contain three single-stranded, negative-sense RNA molecules: S, M and L. Peribunyaviruses have diverse arthropod and vertebrate hosts and are ecologically diverse [117].

##### Bimiti Virus (BIMV)

A new viral agent was retrieved from mosquitoes collected in 1955 in Trinidad after trituration and intracranial inoculation into two-day-old Swiss mice. Evidence of seropositivity from samples collected from residents in close proximity to where mosquitoes were collected was demonstrated: however, no virus was isolated from human subjects [31]. It was not until over four decades later that 17 strains of BIMV were again recovered on the island from mosquitoes collected between 2007 and 2009 [32] (Table 1).

##### Bushbush Virus (BSBV)

A single strain of a new viral agent was recovered from a pool of mosquitoes caught in Trinidad in 1959 (Table 1). Virus was isolated via intracranial inoculation into two-day-old mice; however, attempts to grow the virus in primary hamster kidney cells were unsuccessful. Experimental infectivity studies showed that BSBV was readily maintained in several passages of both *Ae. aegypti* and *Culex quinquefasciatus* mosquitoes [33]. 

##### Cache Valley Virus (CVV)

To date, CVV has only been reported from the islands of Trinidad and Jamaica. In Trinidad, a single strain was identified from a pool of mosquitoes collected in 1958 [34]. Whereas, in Jamaica, multiple isolations occurred between 1962 and 1965 from two different mosquito species [35,36] (Table 1). Virus was isolated via intracranial inoculation into two-day-old mice. A limited epidemiological survey detected antibodies to the virus in humans and animals; however, no associations with clinical cases were reported [48]. Of note, recent studies have determined that the CVV isolates recovered are now considered to be indistinguishable from BeAR7272, which is a Maguari virus isolate [118].

##### Caraparú like Virus (CARV)

During arbovirus studies on the island of Trinidad between 1959 and 1964, multiple isolates of a CARV were recovered from sentinel mice, wild rodents, and different mosquito species using CF and HI tests [37]. Studies conducted between 2007 and 2009 again yielded several isolates identified by CF and molecular methods from various mosquito species [32] (Table 1).

##### Catú Virus (CATUV)

The first strain of CATUV was recovered from cane mice in Trinidad in 1960. This was followed by numerous other isolates retrieved from sentinel mice, mosquitoes, as well as wild rodents [119], and in 1971, from a human [51] (Table 1).

##### Guamá Virus (GMAV)

Guamá virus was first isolated in 1960 in Trinidad from sentinel mice as well as wild rodents. Between 1961 and 1963, additional strains were also recovered from rodents and mosquitoes [37] (Table 1).

##### Kairi Virus (KRIV)

A new viral agent was obtained from a pool of mosquitoes in Trinidad in 1955 via intracranial inoculation of two-day-old mice. Additional isolations were made that year in the same location and again in 1958 at another location, and identification was made by CF, HI, and NT (Table 1). Serological surveys were conducted, with evidence of neutralizing antibodies found in humans and monkeys; however, the interpretation of these results was cautioned [120]. Virus transmission studies were also conducted in Trinidad mosquito species, and it was found that *Aedes scapularis*, *Aedes taeniorhynchus,* and *Mansonia titillans* were competent vectors [38].

##### Lukuni Virus (LUKV)

Two strains of a new viral agent were isolated from three pools of mosquitoes collected in Trinidad in 1955. A third strain was recovered from a pool of mosquitoes collected in 1958 (Table 1). The virus was recovered on intracranial inoculation of two-day-old mice, and it failed to yield positive reactions with other viruses by HI, CF, and NT. Studies demonstrated that LUKV was readily maintained in several passages of both *Ae. aegypti* and *Cx. quinquefasciatus* mosquitoes. Evidence of neutralizing antibodies was found in the sera of humans [33].

##### Manzanilla Virus (MANV)

In 1954, a new viral agent was isolated from a howler monkey in Trinidad. Two-day-old white mice, adult mice, chick embryos, and hamsters were all susceptible to the virus (Table 1). Attempts were made to transmit the virus from mosquitoes but proved unsuccessful. Serological tests of inhabitants of forested areas where the virus was isolated were all negative. However, serological evidence of infection was found in howler monkeys [121].

##### Melao Virus (MELV)

Melao virus was originally identified in Trinidad in 1955 from mosquitoes via intracranial inoculation of two-day-old mice. A combination of NT, HI, and CF tests showed no relationship with other tested viruses. No evidence of neutralizing antibodies was found in residents or monkeys, and no viral isolations were made from the sera of patients with undiagnosed fevers screened between the years 1954 and 1960 [122]. In 2014, MELV was isolated and sequenced using Sanger and next-generation sequencing from school children during routine surveillance of acute febrile illness in Haiti [52] (Table 1).

##### Moriche Virus (MORV)

Only one strain of MORV was recovered during the studies in Trinidad from a pool of mosquitoes collected in 1964 via intracranial inoculation of two-day-old mice [39] (Table 1).

##### Nepuyo Virus (NEPUV)

Between 1957 and 1958, a new viral agent was isolated from a batch of mosquitoes collected in Trinidad that were triturated and subsequently inoculated intracranially into two-day-old mice (Table 1). The new agent showed no reactions with other viruses via CF, HI, and NT. By NT, NEPUV reacted mainly with serum from Murutucu, Maritoba, and Itaqui viruses [123]. These findings were confirmed in studies conducted in Brazil and New York [124]. Evidence of seropositivity was found from sera collected between 1954 and 1960 from residents; however, the actual virus was never retrieved from human samples [123]. Another study from the 1970s found protective sera in two species of bats in Trinidad [125].

##### Oriboca Virus (ORIV)

Three strains of ORIV were isolated from mosquitoes and sentinel mice during arbovirus studies in Trinidad between 1962 and 1963 (Table 1). In the laboratory, *Oryzomys* and *Zygodontomys* were subcutaneously inoculated with the virus, and it was found that it circulated to high titers. In addition, HI, CF, and neutralizing antibodies were detected in their serum [37]. During studies conducted between 2007 and 2009, three isolates of ORIV were retrieved from pools of mosquitoes identified by CF and molecular methods [32].

##### Oropouche Virus (OROV)

In 1955, a new viral agent was isolated from the serum of a 24-year-old male forest worker in Trinidad who presented to a fever clinic. Serological investigations revealed the presence of neutralizing antibodies in forest workers and monkeys. Viral retrieval attempts from mosquitoes and a single species of simulid proved to be unsuccessful. Laboratory infectivity studies showed OROV was retrievable from *Ae. scapularis*, *Aedes serratus*, *Culex fatigans* (now known as *Cx. quinquefasciatus*), and *Psorophora ferox* two weeks post infection [53]. Five years after the discovery of the virus in a human, it was recovered from a pool of mosquitoes collected in 1960. In 2014, OROV RNA was detected in plasma samples from a child in Haiti using an unbiased sequencing approach during monitoring of acute febrile illness in a group of school children (Table 1). Attempts at viral isolation were unsuccessful [52].

##### Pacui Virus (PACV)

During studies in Trinidad, two strains of PACV were recovered from rodents in 1961 [39] (Table 1).

##### Restan Virus (RESV)

In 1963, strains of a new viral agent were retrieved from pools of mosquitoes collected in Trinidad and from the serum of a 16-year-old boy who presented with fever, headache, ague, and pain at the back of his neck (Table 1). All strains were isolated by intracranial inoculation of two-day-old mice, and identification was done by HI, CF, and NT. Infectivity studies showed that the virus circulated in high titers in rodents and could be transmitted by *Ae. aegypti* mosquitoes, supporting the view that rodents could be implicated in the natural cycle of RESV. Sera of residents tested using HI assays only revealed past infections with RESV or closely related viruses [40].

##### Triniti Virus (TNTV)

A new viral agent was obtained from a mixed pool of mosquitoes collected in 1955 in Trinidad. The agent isolated from intracranial inoculation of homogenized mosquitoes into two-day-old mice and the virus yielded no positive reaction when tested with other viruses by HI or NT (Table 1). Evidence of neutralizing antibodies was found in a limited serum survey; however, TNTV was never isolated from human sera or the sera and organs of animals [41].

##### Turlock Virus (TURV)

One strain of this virus was recovered in Trinidad in 1963 from a pool of mosquitoes via intracranial inoculation of two-day-old mice. At the same time, the sera of birds were collected from the area, and only one sample was found to neutralize the virus [42] (Table 1).

##### Wyeomyia Virus (WYOV)

During arbovirus studies conducted from 1953 to 1966 in Trinidad, 28 strains of WYOV were retrieved from various mosquito species via intracranial inoculation of two-day-old mice. Serological evidence of viral infection was found in both humans and birds, but due to cross-reactions in NT, it was not possible to decipher whether these results were specific for WYOV [43]. During studies conducted between 2007 and 2009, two isolates of WYOV were recovered from mosquito pools and were identified by CF and molecular methods [32] (Table 1).

#### 3.3.5. Phenuiviridae

Viruses in this family are enveloped with a tri-segmented, negative-sense RNA genome. They include human, animal, plant, and insect pathogens, which have a diverse range of arthropod vectors [126].

##### Itaporanga Virus (ITPV)

Itaporanga virus was isolated from mosquitoes in Trinidad via intracranial inoculation; however, the exact date of isolation was not reported [44] (Table 1).

#### 3.3.6. Rhabdoviridae

These viruses are ecologically diverse and have negative-sense RNA genomes. They include important pathogens of humans, fish, livestock, or agricultural crops and are transmitted by a range of vectors [127].

##### Aruac Virus (ARUV)

During arboviral studies conducted in the forests of eastern Trinidad in 1955, a new viral agent was isolated from mosquitoes upon intracranial inoculation in two-day-old mice (Table 1). Testing via CF, HI, and NT failed to yield any positive reactions with other viruses. Serological surveys of residents and monkeys all failed to neutralize ARUV in NT assays. In addition, ARUV was not recovered from any human fever cases in Trinidad during the period 1954–1963, nor was it isolated from hundreds of birds and rodents tested during the same time [45].

##### Cocal Virus (COCV)

In 1961, during studies in Trinidad, mites were retrieved from terrestrial rice rats, homogenized and inoculated into two-day-old mice, and COCV was recovered. Three additional isolations were retrieved in 1963 from a pool of mosquitoes, and rodents [128] (Table 1).

#### 3.3.7. Sedoreoviridae

Viruses in this family are nonenveloped with segmented linear dsRNA genomes. They have a wide host range, which includes mammals, arthropods, birds, plants, algae, and crustaceans [129].

##### Bluetongue Virus (BTV)

Many serotypes of BTV are known to be circulating in the Caribbean, although there is little or no evidence that they are causing clinical disease [49,130,131,132,133,134]. Much of the data available on BTV in the Caribbean were collected by a Regional Bluetongue team that studied the disease over eleven countries in the region from the early 1980s to the early 1990s [135]. Circulation of BTV was detected by virus isolation in many Caribbean countries, including Trinidad and Tobago, Barbados, Puerto Rico, Jamaica, Martinique, Guadeloupe, and the Dominican Republic (Table 1). High seroprevalence levels were detected by agar gel immunodiffusion (AGID) in Jamaica (77%), St. Kitts/Nevis (70%), Antigua (76%), St. Lucia (82%), Barbados (61%), Grenada (88%), and Trinidad and Tobago (79%) [134]. In 1981, seroprevalence levels of 80% were detected in cattle in Puerto Rico and the US Virgin Islands (USVI) [136]. A 2015 study in white-tailed deer in the USVI determined the seroprevalence of BTV to be 50% using AGID [137]. Additionally, seroprevalence levels of 99.7% were reported in Cuban cattle [131]. A recent study also confirmed the circulation of multiple BTV serotypes in Trinidad by virus isolation and PCR [138].

##### Epizootic Hemorrhagic Disease Virus (EHDV)

Epizootic hemorrhagic disease virus (EHDV) is an economically important virus that can cause severe clinical disease in deer and, to a lesser extent, cattle. Serum neutralization tests have identified antibodies to EHDV serotype-1 and possibly EHDV serotype-2 to be circulating in cattle in Jamaica, Barbados, Antigua, Grenada, and Trinidad and Tobago [139]. A 2015 study carried out in the USVI using AGID revealed a seroprevalence of 50% in white-tailed deer [137]. Epizootic hemorrhagic disease virus serotype-2 was isolated from biting midges in Puerto Rico [49], and a recent study identified and characterized EHDV serotype-6 in cattle co-infected with BTV in Trinidad and Tobago [140].

##### Ieri Virus (IERIV)

Multiple strains of a new viral agent were isolated from mosquitoes in Trinidad in 1955 via intracranial inoculation of two-day-old mice (Table 1). No positive reactions with other viruses were obtained using HI and CF tests [33].

##### Wad Medani Virus (WMV)

Wad Medani virus was recovered from a pool of larval ticks collected in 1965 in Jamaica [48] (Table 1).

#### 3.3.8. Togaviridae

Viruses in this family are enveloped with single-stranded, positive-sense RNA genomes. Alphaviruses are included in this family, and most are transmitted by mosquitoes [141].

##### Eastern Equine Encephalitis Virus (EEEV)

Evidence of EEEV throughout the Caribbean is much more limited in comparison to that for other encephalitic viruses. Historical records from The Bahamas state than an extensive epizootic outbreak occurred from 1925 to 1927, but no human cases were reported [142]. In Cuba, epizootic outbreaks were reported in horses as early as 1918, though the virus was unknown [143]. Viral outbreaks attributed to EEEV have been reported in studies since the 1930s [144]; however, others have stated that at the time the virus was unknown [143]. Isolation of EEEV was reported as far back as 1943 [145], and during a registered pandemic in 1953 that killed horses and humans [143]. Subsequent recoveries of the virus were made in 1969–1970 from horses [144], and from a pigeon in 1970 [146]. One of the most comprehensive study of EEEV on the island of Cuba occurred between 1972 and 1973 when antibodies were detected in humans, birds, and reptiles, and viral isolations were made from horses, mosquitoes, iguana, and birds [143]. More recent isolations were obtained in 1980 from a rodent and bird, both indigenous to Cuba [147]. In the Dominican Republic, an epizootic outbreak of EEEV in horses was accompanied by cases of human encephalitis between 1948 and 1949, and an isolate was retrieved from the brain of an equine. Clinical equine cases were again reported in 1978, which resulted in further viral isolations. A serological survey showed evidence of recent infections in numerous equines but few humans [148]. In Haiti, neutralizing antibodies to EEEV were identified in one bird in 1972 [86]. Additionally, Madariaga virus (MADV), also known as South American EEEV, which clusters in EEEV lineage II, III, and IV, was reported in 2015. The initial RNA isolate was obtained from cell cultures inoculated with plasma from an infected child, and its identity was confirmed by Sanger sequencing as MADV. The virus was subsequently identified from an additional seven patients from the same study cohort [54].

Jamaica reported that an outbreak of EEE that affected both equines and humans occurred in late 1962, with virus isolations occurring from both species [149,150]. Interestingly though, no serological evidence of EEEV was obtained in a 1956 study [91]. Up to 1970, continued surveillance of EEEV revealed no evidence of its presence among several sentinel flocks of domestic chickens [48]; however, a single positive case was identified in 1973, but no viral isolation was made from postmortem samples [151]. Further serological evidence in humans and birds was provided by surveys conducted throughout the island [48,151,152]. In Trinidad, serological evidence for EEEV preceded detection of the live viral agent on the island of Trinidad. In 1954, serum collected from a donkey tested positive for antibodies to EEEV [73], but it was not until 1959 that the first strain of EEEV was isolated from a pool of mosquitoes [153]. Researchers went on to isolate two additional strains of EEEV later that year from pools of mosquitoes. It would not be until March 1970 that EEEV would be isolated from a human subject in Trinidad [55] (Table 1).

##### Madariaga Virus

See section on (EEEV).

##### Mayaro Virus (MAYV)

In 1954, a blood sample taken from a febrile forest worker in Trinidad yielded a new viral agent [56], and MAYV was isolated from the blood of four other patients with febrile illness during that same year. A serological survey conducted on the population found that fifty percent of the older people in the southeastern part of the island had neutralizing antibodies to MAYV [154]. It was not until 1957 that MAYV was retrieved from a pool of mosquitoes via intracranial inoculation of two-day-old mice. The virus was indistinguishable from the other strains isolated in Trinidad by CF, HI, and NT [155]. During the chikungunya outbreak in Haiti in 2014, identification of arboviral dual infections revealed a patient infected with both chikungunya virus (CHIKV) and MAYV. The virus was identified using Sanger sequencing after infected Vero E6 cell cultures displayed cytopathic effects that were inconsistent with a CHIKV infection [57]. Three other cases of MAYV were subsequently reported from the same cohort of children [156]. Mayaro virus was also detected in a child in Haiti in 2015 during surveillance of a cohort of school children with an acute undifferentiated febrile illness [58] (Table 1).

##### Mucambo Virus (MUCV)

See section on VEEV.

##### Una Virus (UNAV)

Una virus was isolated for the first time in Trinidad in 1959 from a pool of mosquitoes via intracranial inoculation of two-day-old mice. Additional strains were identified between 1960 and 1962 from mosquitoes and sentinel mice [39] (Table 1).

##### Venezuelan Equine Encephalitis Virus (VEEV)

An outbreak of VEEV occurred in Trinidad in 1943, and humans and equines were affected. Virus was isolated from equines and mosquitoes by intracranial inoculated of homogenized material in two-day-old mice [157]. Over a decade following the first outbreak, arbovirus studies conducted from 1959 to 1964 yielded 262 isolations of VEEV. With the exception of 1964, VEEV was isolated in all years of the study period, and seventeen species were found to be infected with VEEV. Antibodies were found equally among the wild rodents, further supporting the evidence from the virus isolations [37]. Recently, a study on sylvatic mosquito-borne viruses in Trinidad conducted between 2007 and 2009 recovered Mucambo virus (VEE complex subtype IIIA) from mosquitoes. Phylogenetic analysis of these newly isolated strains with older strains from Trinidad and Brazil suggested that there is a geographic structure within Trinidad that is consistent with enzootic maintenance in localized rodent populations with limited dispersal and range [32]. Another study detected VEEV antibodies in horses using HI and epitope-blocking enzyme immunoassay [98], indicating a low-level circulation of VEEV in Trinidad horses. Another study conducted between 2006 and 2008 revealed evidence of seropositivity in Trinidad bats for VEEV [97] (Table 1).

##### Western Equine Encephalitis Virus (WEEV)

Few reports of WEEV have emerged from islands in the Caribbean. Isolation of WEEV occurred in Cuba in 1970 from a sick pigeon [144] (Table 1). In Curaçao, serological evidence of WEEV in humans was reported from a study conducted in 1953 [75], but these findings were not supported in a 1963 study [69]. Serological evidence of the virus was also found in birds and bats in Haiti in 1972 [86].

**Table 1:** Isolation of arboviruses or detection of nucleic acids in the islands of the insular Caribbean.

Arbovirus	Island	Date	Source	References
Flaviviridae				
Ilhéus virus	Trinidad	1954	Mixed pool of mosquitoes, *Psorophora* spp.,	[74]
		1955	Mixed pool of mosquitoes, *Homo sapiens*	[50,74]
		1956	Mixed pool of mosquitoes, *Psorophora ferox*	[74]
		1966	*Homo sapiens*	[50]
		1967	*Homo sapiens*	[50]
St Louis encephalitis virus	Haiti	1955	*Butorides virescens*	[85]
	Jamaica	1962	*Culex nigripalpus*, *Mimus polyglottos*	[87,89]
		1963	*Culex nigripalpus*	[88]
	Trinidad	1955	*Culex coronator*, *Culex caudelli*, *Psorophora ferox*	[93]
		1956	*Leptotila verreauxi*	[94]
		1958	*Ramphocelus carbo*, *Turdus fumigatus*, *Manacus manacus*, *mosquitoes*, *Homo sapiens*	[95,96]
			*Pipra erythrocephola*, *mosquitoes*	[95]
		1960–1962	*Aedes scapularis*, *Culex caudelli*, *Culex coronator*, *Culex declarator*, *Culex nigripalpus*, *Culex spissipes*, *Culex taeniopus*, *Psorophora ferox*	[95]
Spondweni virus	Haiti	2016	*Culex quinquefasciatus* ^a^	[30]
West Nile virus	Puerto Rico	2006	*Gallus gallus*, *Culex nigripalpus*, *Culex bahamensis*	[104]
Nairoviridae				
Estero real virus	Cuba	1980	*Ornithodoros tadaridae*	[46]
Hughes virus	Cuba	1979	*Ornithodoros denmarki*	[115]
		1980	*Ornithodoros denmarki*	[116]
	Trinidad	1962	*Ornithodoros capensis*, *Ornithodoros denmarki*, *Sterna fuscata*	[114]
Soldado virus	Trinidad	1963	*Ornithodoros capensis*	[47]
Peribunyaviridae				
Bimiti virus	Trinidad	1955	*Culex* spp., *Culex spissipes*	[31]
		2007–2009	*Culex portesi*	[32]
Bushbush virus	Trinidad	1959	*Culex amazonensis*	[33]
Cache Valley virus ^b^	Jamaica	1962	*Anopheles grabhami*	[35]
		1963	*Aedes taeniorhynchus*	[35]
		1965	*Anopheles grabhamii*, *Aedes taeniorhynchus*	[35,36]
	Trinidad	1958	*Aedes scapularis*	[34]
Carapurú like virus	Trinidad	1959–1964	*Culex portesi*, *Culex accelerans*, *Culex amazonensis*, *Culex coronator*, *Culex spissipes*, *Culex nigripalpus*, *Culex vomerifer*, *Wyeomyia medioalbipes*	[37]
		2007–2009	*Culex portesi*, *Culex vomerifer*, *Culex pedroi*	[32]
Catú virus	Trinidad	1960–1964	*Zygodontomys brevicauda*, *Oryzomys laticeps*, *Culex portesi*, *Culex amazonensis*, *Culex crybda*	[119]
		1971	*Homo sapiens*	[51]
Guama virus	Trinidad	1960–1963	*Heteromys anomalus*, *sentinel mice*, *Oryzomys*, *Zygodontomys*, *Heteromys*, *Proechimys*, *Culex amazonensis*, *Culex portesi*, *Mansonia* spp.	[37]
Kairi virus	Trinidad	1955	*Aedes scapularis*, *Psorophora ferox*, *Culex spissipes*, *Wyeomyia aporonoma*	[38]
		1958	*Wyeomyia ypsipola*	[38]
Lukuni virus	Trinidad	1955	*Aedes scapularis*	[33]
Manzanilla virus	Trinidad	1954	*Alouatta seniculus insularis*	[121]
Melao virus	Haiti	2014	*Homo sapiens*	[52]
	Trinidad	1955	*Aedes scapularis*	[122]
Moriche virus	Trinidad	1964	*Culex amazonensis*	[39]
Nepuyo virus	Trinidad	1957–1958	*Culex accelerans*	[123]
Oriboca virus	Trinidad	1962–1963	*Culex portesi*, Sentinel mice	[37]
		2007–2009	*Culex portesi*	[32]
Oropouche virus	Haiti Trinidad	2014 1955	*Homo sapiens* ^a^ *Homo sapiens*	[52] [53]
		1960	*Mansonia venezuelensis* ^b^	[53]
Pacui virus	Trinidad	1961	*Zygodontomys brevicauda*, *Oryzomya laticeps*	[39]
Restan virus	Trinidad	1963	*Culex portesi*	[40]
Triniti virus	Trinidad	1955	Mixed pool containing *Trichoprosopon digitatum*, *Trichoprosopon theobaldi*, *Trichoprosopon longipes*	[41]
Turlock virus	Trinidad	1963	*Culex declarator*	[42]
Wyeomyia virus	Trinidad	1953–1966	*Aedes scapularis*, *Culex amazonensis*, *Limatus durhamii*, *Limatus flavisetosus*, *Trichoprosopon longipes*, *Psorophora albipes*, *Psorophora ferox*	[43]
		2007–2009	*Trichoprosopon digitatum*, *Wyeomyia* spp.	[32]
Phenuiviridae				
Itaporanga virus	Trinidad	Not stated	*Culex eastor*	[44]
Rhabdoviridae				
Aruac virus	Trinidad	1955	*Trichoprosopon theobaldi*, *Psorophora ferox*	[45]
Cocal virus	Trinidad	1961	*Orzomys laticeps velutinus*, *Culex portesi*, *Heteromys anomalis*	[128]
Sedoreoviridae				
Bluetongue virus	Barbados	1987–1992	Ovine	[49,133]
	Dominican Republic	1987–1992	Bovine	[49,133]
	Guadeloupe	2006	Bovine	[135]
	Jamaica	1987–1992	Bovine	[49,133]
	Martinique	2006	Bovine	[132]
	Puerto Rico	1987–1992	Bovine, *Culicoides pusillus*	[49,133]
	Trinidad	1987–1992	Bovine	[49,133]
		2017	Bovine	[138]
Epizootic haemorrhagic virus	Puerto Rico	1987–1992	*Culicoides pusillus*	[49]
	Trinidad	2019	Bovine	[140]
Ieri virus	Trinidad	1955	*Psorophora albipes*, *Psorophora ferox*	[33]
Wad Medani virus	Jamaica	1965	*Amblyomma cajennense*	[48]
Togaviridae				
Eastern equine encephalitis virus	Cuba	1943	Equine	[145]
		1953	Equine	[143]
		1969–1970	Equine	[144]
		1970	*Columba livia*	[146]
		1972	Equine, *Aedes taeniorhynchus*, *Cyclura macleaya*, *Egretta thula*, *Butorides virescens*, *Guara alba*, *Buteogallus gunlachi*, *Tringa solitaria*, *Saurothera merlini*, *Tolmarchus caudifasciatus*, *Corvus nasicus*	[143]
		1980	*Capromys pilorides*, *Dumetella carolinensis*	[147]
		1949	Equine	[158]
	Dominican Republic	1978	Equine	[148]
	Jamaica	1962	Equine, *Homo sapiens*	[150]
	Trinidad	1959	*Culex nigripalpus*, *Culex taeniopus*	[153]
		1970	*Homo sapiens*	[55]
Madariaga virus	Haiti	2015	*Homo sapiens*	[54]
Mayaro virus	Haiti	2014	*Homo sapiens*	[57]
		2015	*Homo sapiens*	[58]
	Trinidad	1954	*Homo sapiens*	[56]
		1957	*Mansonia venezuelensis* ^c^	[155]
Una virus	Trinidad	1959	*Psorophora ferox*	[39]
		1960	*Culex ybarmis*, Sentinel mice	[39]
		1962	*Aedes serratus*	[39]
Venezuelan equine encephalitis	Trinidad	1943	*Mansonia titillans*, Equine	[157]
		1959–1963	*Culex portesi*, *Aedes serratus*, *Culex amazonensis*, *Limatus flavisetosus*, *Mansonia*, *titillans*, *Mansonia venezuelensis* ^c^, *Psorophora ferox*, *Wyeomyia medioalbipes*, *Orozymus*, *Zygodontomys*, *Heteromys*, Sentinel mice	[37]
Mucambo virus	Trinidad	2007–2009	*Culex portesi*	[32]
Western equine encephalitis	Cuba	1970	*Columba livia*	[144]

^a^ Nucleic acids detected by sequencing only; ^b^ Studies now show isolates indistinguishable from BeAR7272, which is a Maguari virus isolate; ^c^ Now known as *Coquillettidia venezuelensis.*

## 4. Discussion

### 4.1. Distribution of Arboviruses in the Caribbean

This review illustrates the vast diversity of arboviruses, which spans eight families, that have been identified in the Caribbean region. From the data presented, it is obvious that there is strong heterogeneity in the distribution of arboviruses. This can be a reflection of surveillance efforts and the number and biodiversity of vectors and hosts, among other factors. Although serological studies were conducted on many islands, interpretations in many cases were inconclusive. This is due to the possibilities of cross-reactivity amongst arboviral families; therefore, there is only weak evidence for the actual presence of some arboviruses [159]. As such, Table 1 only lists arboviruses where virus isolations occurred, or identification was made using molecular methods.

Trinidad, the southernmost island of the region, possesses by far the lion’s share of arbovirus findings in the entire insular Caribbean. The Trinidad Regional Virus Laboratory (TRVL), which was established in 1952 by the Rockefeller Foundation, embarked on a colossal surveillance project that yielded the vast majority of arboviruses found on the island [160]. Additionally, Trinidad was once attached to the South American continent and only separated approximately 10,000 years ago [161]. As such, the island possesses the most diverse flora and fauna found anywhere in the Caribbean and is home to over 170 species of mosquitoes [162] and over 45 species of ticks [163]. In contrast, the smaller islands of the Lesser Antilles are underrepresented in this review. While the diversity of vectors is significantly less [162,164,165], it is evident that the lack of conclusive information from these islands is directly related to inadequate capacities in both vector and viral surveillance. In fact, most of the evidence reported from these islands was a result of single serological studies conducted by the TRVL in the 1950s and 1960s.

As seen in Table 2, studies from Trinidad show a preponderance of viruses in the family *Peribunyaviridae*. Interestingly, only two other islands, Jamaica and Haiti, reported viruses from this family. In both instances, discoveries were made during ongoing surveillance efforts with international collaborators. Work in Jamaica was done in conjunction with the TRVL, and Haiti, which has recently emerged as the island with the second largest number of identified arboviruses, has also been supported by international partners. These discoveries highlight the importance of sustained collaborative surveillance efforts on all Caribbean islands.

### 4.2. Resurgence of Arboviral Threats

Evidence of viruses belonging to the *Flaviviridae* and *Togaviridae* families was more widely recorded across the islands. Historical records show that SLEV, EEEV, VEEV, WEEV, and more recently, WNV are among the most prominent arboviruses in these families. It is noteworthy that disease outbreaks caused by these viruses have either not been reported for decades or, as in the case of WNV, not recorded. There is an urgent need to investigate as this could be a result of a multiplicity of factors, including under-reporting, lack of diagnostic capabilities, and possible cross-protection from prior infections with other related arboviruses [166]. Adding to this, although there have been numerous reports of arboviral infections in migratory birds throughout the region, the role that they play in the dispersal of arboviral pathogens in the Caribbean is yet to be completely understood.

Records from the region also demonstrate that the identification of uncommon arboviruses, such as those with single isolations, does not necessarily represent static events. Arboviral cycles are dynamic, and viruses possess the propensity to reappear under favorable conditions. The recent identification of Melao virus from five children in Haiti in 2014 [52], almost six decades after its discovery in a Trinidadian forest in 1955 [122], lucidly illustrates this fact, along with the re-isolation of Bimiti virus in Trinidad [32] four decades after its original discovery on the island in 1955 [31]. Arboviral resurgence can also be facilitated by a lack of effective vector control, which has historically been an issue within the Caribbean [167]. This was evident in the 1970s when the discontinuation of many vector control programs led to mosquito reinfestations, with arbovirus epidemics swiftly following [168]. Today, the added issue of insecticide resistance has further compounded vector control challenges [169,170]. However, newer strategies such as the use of biological agents, release of insects carrying a dominant lethal gene, and the sterile insect technique are viable options that should be explored [171,172,173].

Furthermore, one of the less extensively studied drivers of arbovirus resurgences, which remains largely unexplored in the region, is the genetic determinants of the viruses themselves. Reports from outside the Caribbean showed that mutations in the envelope proteins of Zika virus facilitated viral fitness [174,175] and mutations in the NS3 helicase and envelope protein of WNV resulted in increased virulence in the American crow and reduced extrinsic incubation periods in *Culex* mosquitoes [176,177]. Added to this, the loss of immunity in subsets of a population can also facilitate resurgence. The re-introduction of DENV-2 in certain countries in the Americas, where the majority of cases occurred in children under the age of 15 years who lacked previous exposure to this serotype, exemplified this point [178].

Lastly, both MAYV and OROV are at high risk for resurgence/emergence within the region due to their viral structures, ever expanding geographic distribution, and involvement in outbreaks in multiple countries [179,180]. Although the main vector for MAYV is mosquitoes of the genus *Haemagogus,* it can be vectored by other mosquitoes such as *Aedes* spp. *Culex* spp., *Sabethes* spp., *Psorophora* spp., and *Coquillettidia* spp. Of note, it has been demonstrated that both *Ae. aegypti* and *Ae. albopicus* can vector MAYV, the latter of which keeps expanding its geographic range. Since its discovery, MAYV has been identified in countries throughout Central and South America in addition to Haiti and Trinidad and Tobago in the insular Caribbean. Similarly, multiple potential vectors of OROV have been identified, including culicoides and mosquitoes belonging to the genera *Aedes*, *Culex*, *Psorophora,* and *Coquillettidia*. Interestingly, *Ae. aegypti* and *Ae. albopictus* are both included among these. OROV has also been identified in multiple countries in Central and South America and Haiti and Trinidad and Tobago in the Caribbean [181].

### 4.3. The Need for Increased Regional Capacity

The Caribbean faces many challenges when approaching the problem of arboviruses. Due to the multifactorial drivers of arbovirus emergence and resurgence, focusing on any one aspect will not solve the problem altogether. Rather, a One Health approach that addresses multiple, if not most, factors would likely produce a more pronounced, lasting positive outcome. There is a great need for stable, well-funded, ongoing research initiatives that continuously survey vectors, animals, and humans for identification and characterization of arboviruses in the region. Because of limited resources, this can only be accomplished with the help of international collaborators. Currently, however, at least screening for viruses at high risk of resurgence/emergence, such as MAYV and OROV, may be beneficial. Building regional capacities to undertake these types of studies, such as biosafety level 3 laboratories and core sequencing facilities, would greatly reduce the complexities for such investigations. While the capacity for viral sequencing within the Caribbean has undoubtedly increased due to the COVID-19 pandemic, the lack of infrastructure and skilled technicians throughout the region and the financial costs involved with the use of these technologies will continue to negatively affect research efforts. Lastly, entomological education and training should be prioritized to ensure there is a robust capacity to identify new vector species, as most islands either lack recent baseline data or possess none. The first reports of *Aedes vittatus* in the Americas in the Dominican Republic and Cuba [182,183,184] and the expansion of *Ae. albopictus* into Jamaica [185], all occurring in recent years, are salient examples of this need.

### 4.4. Limitations of the Study

This review was limited by the scarcity of published primary studies from many islands and the paucity of recent information available on most of the arboviruses discussed. Additionally, as the majority of the information pertaining to the lesser-known arboviruses in the region is dated, articles published in now obsolete journals may have been missed, resulting in information gaps.

## 5. Conclusions

As the planet continues to warm and vector species expand their geographic habitats, knowledge of the lesser-known arboviruses become important, as well as understanding the factors that facilitate their emergence and resurgence in the Caribbean. The One Health approach can lead to sustained solutions for controlling and suppressing arboviral diseases. However, bridging essential research gaps and obstacles is critical to curb further spread and dissemination within the insular Caribbean and globally.

## Figures and Tables

**Table 2:** Distribution of arboviruses in the islands of the Caribbean.

Country	*Asfaviridae*	*Flaviviridae*					*Nairoviridae*			*Peribunyaviridae*																			*Phenuiviridae*	*Rhabdoviridae*		*Sedoreoviridae*				*Togaviridae*						
	ASFV	ILHV	JEV	SLEV	SPONV	WNV	ERV	HUGV	SOLV	BIMV	BSBV	CVV ^a^	CARV	CATUV	GMAV	KRIV	LUKV	MANV	MELV	MORV	NEPUV	ORIV	OROV	PACV	RESV	TNTV	TURV	WYOV	ITPV	ARUV	COCV	BTV	EHDV	IERIV	WMV	EEEV	MADV ^b^	MAYV	MUCV ^c^	UNAV	VEEV	WEEV
Antigua and Barbuda		X ^§^		X ^§^																												X	X									
Aruba				X ^§^																																						
Bahamas						X																														X						
Barbados				X ^§^																												X	X									
British Virgin Islands						X																																				
Cayman Islands						X																																				
Cuba	X			X		X	X	X																								X				X						X
Curaçao		X ^§^	X ^§^	X																																						X ^§^
Dominican Republic	X			X		X																										X				X						
Grenada		X ^§^		X ^§^																												X	X									
Guadeloupe						X																										X	X									
Haiti	X			X	X	X													X				X													X	X	X				X
Jamaica				X		X						X																				X	X		X	X						
Martinique																																X	X									
Montserrat				X ^§^																																						
Puerto Rico				X		X																										X										
St. Eustatius						X																																				
St. Kitts and Nevis						X																										X	X									
St Lucia		X ^§^																														X	X									
St. Vin. and the Gren.		X ^§^		X ^§^																																						
Trinidad and Tobago		X		X		X		X	X	X	X	X	X	X	X	X	X	X	X	X	X	X	X	X	X	X	X	X	X	X	X	X	X	X		X		X	X	X	X	
USVI																																X	X									

African swine fever virus (ASFV), Ilhéus virus (ILHV), Japanese encephalitis virus (JEV), St Louis encephalitis virus (SLEV), Spondweni virus (SPONV), West Nile virus (WNV), Estero Real virus (ERV), Hughes virus (HUGV), Soldado virus (SOLV), Bimiti virus (BIMV), Bushbush virus (BSBV), Cache Valley virus (CVV), Caraparú virus (CARV), Catú virus (CATUV), Guamá virus (GMAV), Kairi virus (KRIV), Lukuni virus (LUKV), Manzanilla virus (MANV), Melao virus (MELV), Moriche virus (MORV), Nepuyo virus (NEPUV), Oriboca virus (ORIV), Oropouche virus (OROV), Pacui virus (PACV), Restan virus (RESV), Triniti virus (TNTV), Turlock virus (TURV), Wyeomyia virus (WYOV), Itaporanga virus (ITPV), Aruac virus (ARUV), Cocal virus (COCV), bluetongue virus (BTV), epizootic hemorrhagic disease virus (EHDV), Ieri virus (IERIV), Wad Medani virus (WMV), eastern equine encephalitis virus (EEEV), Madariaga virus (MADV), Mayaro virus (MAYV), Mucambo virus (MUCV), Una virus (UNAV), Venezuelan equine encephalitis virus (VEEV), western equine encephalitis virus (WEEV). ^a^ Studies now show isolates indistinguishable from BeAR7272 which is a Maguari virus isolate; ^b^ Member of EEEV complex; ^c^ Member of VEEV complex; ^§^ inconclusive serological evidence.

## Data Availability

Not applicable.
